# A Hybrid Simulation–Physical Data-Driven Framework for Occupant Injury Prediction in Vehicle Underbody Structures

**DOI:** 10.3390/s26020380

**Published:** 2026-01-07

**Authors:** Xinge Si, Changan Di, Peng Peng, Yongjian Zhang, Tao Lin, Cong Xu

**Affiliations:** Department of Mechanical Engineering, Nanjing University of Science and Technology, Nanjing 210094, China

**Keywords:** finite element simulation, occupant injury mitigation, wavelet domain, conditional generative adversarial network, Gaussian process regression, vehicle underbody structure

## Abstract

One major challenge in optimizing vehicle underbody structures for blast protection is the trade-off between the high cost of physical tests and the limited accuracy of simulations. We introduce a predictive framework that is co-driven by limited physical measurements and systematically augmented simulation datasets. The main problem arises from the complex components of blast impact signals, which makes it difficult to augment the load signals for finite element simulations when only extremely small sample sets are available. Specifically, a small-scale data-augmentation model within the wavelet domain based on a conditional generative adversarial network (CGAN) was designed. Real-time perturbations, governed by cumulative distribution functions, were introduced to expand and diversify the data representations for enhanced dataset enrichment. A predictive model based on Gaussian process regression (GPR) that integrates physical experimental data with augmented data wavelet characteristics is employed to estimate injury indices, using wavelet scale energies reduced via principal component analysis (PCA) as inputs. Cross-validation shows that this hybrid model achieves higher accuracy than using simulations alone. Through the case study, the model demonstrates that increased hull angle and depth can effectively reduce occupant injury.

## 1. Introduction

With the recurring occurrence of terrorist attacks and accidental explosions, risks to public safety and transportation systems have become increasingly prominent. To conduct post-event assessments, optimize vehicle structures, and support emergency decision-making more effectively under extreme shock conditions, it is crucial to predict occupant responses rapidly and reliably. Acceleration signals captured at a limited number of external measurement points on the vehicle contain important information regarding the propagation of shock energy and the associated structural dynamics. However, real-world explosion experiments are highly costly and hazardous, and are further constrained by ethical and practical limitations, resulting in very limited available data. This scarcity often leads prediction models to suffer from overfitting and inadequate generalization. Considering that vehicle response signals under explosive loading exhibit abrupt, multi-component, and complex transient characteristics, this study proposes an injury prediction approach based on data augmentation for such shock-type signals.

To eliminate human subject risk during the protective experimental process, the Hybrid III 50th ATD is widely used as a dummy to test for damage to occupants. It is currently a mature means for explosion injury testing. Many scholars have studied the response of Hybrid III under impact. Kristin et al. compared the modal frequencies and short-duration impact responses of Hybrid III and NOCSAE headforms to assess their biofidelity and highlight the importance of headform choice under high-frequency impacts [1]. Li et al. used the Hybrid III 50th percentile male dummy for occupant kinematics and injury metric validation in vehicle crash scenarios [2]. Albert et al. compared the whole-body biofidelity of Hybrid III and THOR in sled tests, including dynamic response, chest deformation, and reaction forces [3].

However, the Hybrid III ATD’s prohibitive acquisition cost and vulnerability under blast loads severely restrict the ability to obtain statistically significant sample sizes.

A finite element (FE) dummy model has been developed to visualize occupant force distribution and reduce experimental costs. The widely used Hybrid III FE model was developed by LSTC and the University of Washington. Subsequent studies have refined the material characterization of this model under high-velocity impact scenarios by incorporating strain-rate sensitivity. Lou enhanced the mechanical fidelity of the lumbar spine by employing a stiffer viscoelastic material formulation [4]. Similarly, Zhu validated the dynamic properties of several soft tissues, including those of the pelvis, lower limbs, heel padding, and skin, under elevated strain rates [5]. Experimental work by Suhaimi further confirmed the accuracy of the improved Hybrid III 50th percentile FE model showing strong agreement with physical test data. Quantitative comparisons indicated correlation scores ranging from 0.653 to 0.901, while peak response deviations were within 1.5% to 12.7% [6,7]. Zheng used the EARTH metric for automatic model calibration to improve prediction accuracy [8]. Zheng combined dynamic response error evaluation with Bayesian inference to refine model parameters [9]. In terms of applications, Palta used Hybrid III 50th FE model to simulate crashes with airbags/seatbelts and assess occupant injury [10]. Maier combined multibody dynamics and FE to model rider-motorcycle interactions [11]. Shi compared Hybrid III FE and human HBM models to study head, neck, and torso responses [12].

However, existing FE model optimizations are often performed under specific working conditions. Edri investigated the injury risks from blast waves generated by explosions near military trenches, employing Computational Fluid Dynamics (CFD) simulations [13]. In practical applications, significant discrepancies may exist between the finite element analysis (FEA) conditions and the specific blast scenarios, which can lead to distorted results in varying blast environments.

Data augmentation is essential when experimental data are limited. Li et al. applied a WGAN-GP-based data augmentation strategy to address the severe class imbalance in crash injury severity prediction, significantly improving the prediction performance for rare severe injury cases [14]. Yin et al. proposed a WTSS-based framework to generate large-scale and credible virtual war trauma data under extremely limited real data conditions [15]. Vahid et al. proposed a transformer-based time-series WGAN with gradient penalty for synthetic acceleration data [16]. Liu et al. used Monte Carlo sampling and 1000 automated simulations to study how vehicle front-end structures affect e-bike rider injuries [17]. Nonetheless, existing data augmentation methods are generally limited to either purely data-driven generative approaches or purely FE-based simulation approaches.

In contrast, the proposed approach adopts a hybrid strategy in which data-driven load generation is coupled with physics-based finite element analysis, thereby combining the flexibility of GANs with the reliability of physical modeling.

In the field of data-driven modeling for safety-critical scenarios, several recent studies have made important contributions, for example: Zhou et al. using data-driven modeling, demonstrated that automated vehicles could avoid 60.91% of reconstructed crashes and reduce injuries in the remaining cases, highlighting their superior safety over human drivers [18]. Yang et al. analyzed vehicle trajectories at Changsha Airport, showing that frequent lane changes and overlapping paths drive traffic conflicts, and offering guidance for safer, more efficient airport pick-up areas [19]. Mostafa et al. developed an AI-driven machine learning framework using over 2.26 million records, showing that integrating human, crash, and vehicle factors with advanced feature selection and ensemble modeling improves crash severity prediction and supports data-driven traffic safety [20]. Vicent et al. developed a three-stage deep learning framework, including preprocessing, feature importance analysis, and 2D-CNN modeling, to predict whether traffic accidents require medical assistance, demonstrating superior accuracy across datasets from six cities [21]. Carlo et al. proposed a generative artificial intelligence-based approach to augment human biomechanical data under limited data conditions [22]. These studies primarily rely on single data-driven modeling approaches, typically trained on one type of data source. They often lack the ability to fully exploit complementary information from heterogeneous data.

The main contributions of this study can be summarized as follows:

- A novel framework is developed to improve occupant injury prediction under underbody blast loading in an extremely low-sample regime by effectively combining scarce physical experimental data with augmented simulation data. Specifically, a wavelet-domain CGAN is employed to generate realistic load perturbations, which are subsequently applied to FE model to produce physically consistent injury responses, thereby expanding and diversifying the very limited physical dataset.
- Enriched experimental and simulated data are processed through PCA on wavelet scale energies and used as inputs to a GPR model, achieving higher predictive accuracy than traditional finite element simulations.
- The proposed methodology enables reliable injury prediction for both V-hull and flat-hull vehicle underbody structures and can be extended to other impulse load testing and safety-critical applications.

The rest of this paper is structured as follows. Section 2 presents the workflow of the digital–physical data-driven framework, along with, data augmentation approach, and prediction model. Section 3 provides experimental and numerical simulation results, along with an evaluation of the predictive abilities of both the numerical simulation and the prediction model. Section 4 presents a set of simulation cases to demonstrate the relationship between the armored vehicle underbody structure and occupant injuries using the trained prediction model. Section 5 discusses the current limitations of the proposed method and outlines directions for future work. Finally, the conclusions are presented in Section 6.

## 2. Method

### 2.1. Method Workflow

**Figure 1:**
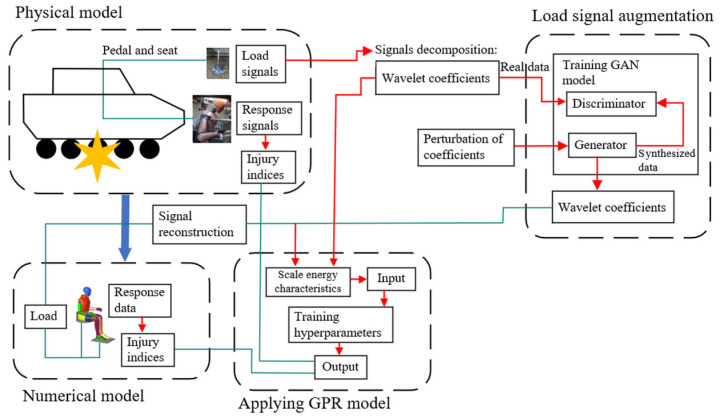
The workflow of the proposed method (The green arrows indicate the data flow between different modules, the red arrows represent the data reconstruction processes, and the blue arrow denotes the main workflow linking the physical experiments with the numerical modeling).

The framework proposed in this study presents a prediction methodology that integrates physical test data with augmented simulation data for the underbody structural optimization of armored vehicles, illustrated in Figure 1. In the physical model, the vehicle underbody is subjected to blast impact experiments, from which load signals and response signals of the occupant are recorded, and corresponding injury indices are calculated. For the data-driven model, the test load signal is first decomposed into multi-scale coefficients using the wavelet transform. A small-scale data-augmentation model within the wavelet domain, based on a conditional generative adversarial network (CGAN), was designed. Through adversarial training of the generator and discriminator with perturbations applied based on the cumulative distribution function, the network is optimized. The signals generated by the augmentation model are reconstructed and applied as loading inputs to the optimized Hybrid III 50th percentile FE model. The numerical model produces response data, which are subsequently converted into the corresponding injury indices. In this way, an augmented dataset of load signals and injury indices is obtained.

The data from the tested physical model and the augmented digital model are then integrated to establish a mapping between load signal characteristics and injury indices. Wavelet energies at each scale are first computed from the multi-source coefficients, and PCA is subsequently applied to extract characteristics and reduce dimensionality. These characteristics are then fed into a GPR model, which predicts the corresponding injury indices. By optimizing the hyperparameters of the GPR model, a coupling between the physical and data-driven models is achieved. Finally, the trained GPR model is incorporated into the optimization stage to determine the optimal geometry and design parameters of the armored vehicle’s underbody structure. The model provides predictive injury indices under various blast impact conditions.

We next detail the structure and design rationale of each module in the process.

### 2.2. Data Augmentation Method

#### 2.2.1. Data-Augmentation Model

**Figure 2:**
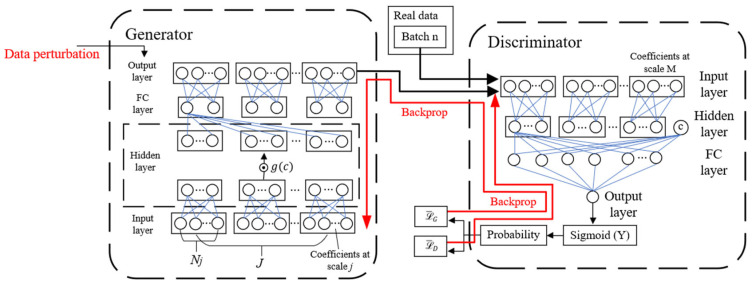
Architecture of data-augmentation model (The symbol c in discriminator denotes the conditional input, and g(c)  in generator represents the gating function conditioned on c).

To enable signal augmentation with extremely limited samples while preserving features across all scales, a data-augmentation model in the wavelet domain based on CGAN was developed. The principle of GAN is to optimize the generator and discriminator through adversarial training [23,24]. In this work, a multi-channel fully connected neural network architecture is employed for multiple frequency components [25]. The network structure is illustrated in Figure 2. First, the signal is decomposed into multiple scale coefficients using the DB4 wavelet, with each scale coefficient treated as a separate channel for the network. The hidden layers are designed for feature extraction from the input coefficients, while the fully connected (FC) layers establish the mapping from the features at different scales. To prevent overfitting under extremely small sample conditions, a small number of nodes are used in the hidden and fully connected layers. A conditional input *c* is introduced to the gated hidden layer in order to control the energy of the generated output in the generator. In the discriminator, *c* is incorporated into the hidden layer. To stabilize training, the conditional vector *c* is obtained by normalizing the amplitude of each real sample. In each training iteration, two sets of data are sampled from the real dataset to form a batch for training.

The Daubechies 4 (DB4) wavelet [26] is characterized by its compact support and short filter length, which provides excellent temporal localization for accurately capturing transient events such as impacts and shocks. Concurrently, it offers a sufficient frequency resolution to distinguish constituent frequency components. This favorable time-frequency characteristic makes DB4 particularly suitable for decomposing vehicle explosion shock signals. The original signal is first decomposed using the DB4 wavelet transform:

(1) $$x_{j}(t)=\varphi_{j,t}$$

where $\varphi_{j,t}$ is the wavelet coefficients vector at scale *j* of *t*-th.

To enhance the model’s stable output, *x* is normalized to $\hat{x}$.

$E_{t}$ denotes the total energy of the *t*-th signal, which reflects the overall signal intensity.

$$E_{t}=\sum_{j}\sum_{n=1}^{N_{j}}\varphi_{j,t}^{2}(n)$$

where $\;N_{j}=N/2^{j}$ is the length of the coefficients at scale *j*; *N* is the length of the original signal.

The condition c is defined based on the normalized total energy of each signal:

(2) $$c=\frac{E_{t}-E_{min}}{E_{max}-E_{min}}$$

This normalization is applied after data augmentation to avoid numerical dominance caused by large-amplitude samples, while preserving the relative variations introduced by amplitude-based augmentation.

$E_{min}$ and $E_{max}$ are the minimum and maximum energy of the signal, respectively.

Forward propagation refers to the process by which data passes through the network to produce output. The following are forward propagation from the input layer to the hidden layer in the generator:

(3) $$X_{im}^{Hidden,G}=\sum_{j=1}^{N}W_{jim}^{\left(1\right),G}{\hat{X}}_{jm}^{Input,G}+b_{im}^{\left(1\right),G}$$

$X_{im}^{Hidden,G}$ represents the i-th node in the hidden layer in m channel in the generator; $W_{ijm}^{\left(1\right)}$ represents the weight connecting the j-th node of the previous layer to the i-th node of first layer in m channel in the generator; $b_{im}^{\left(1\right)}$ represents the bias of the i-th node of first layer in m channel in the generator. For the rest of this section, the same naming convention is applied.

The hidden layer is gated by the condition *c*.

(4) $$g\left(c\right)=Sigmoid(W_{g}c+b_{g})$$

(5) $${X_{im}^{Hidden,G}}^{\prime }=X_{im}^{Hidden,G}⊙g\left(c\right)$$

Before feeding into the FC layer, the hidden layer needs to be flattened. The flattened hidden layer is given by:

(6) $${X_{j}^{Hidden,G}}^{\prime }⟶{X_{im}^{Hidden,G}}^{\prime },\;j=i+(m-1)N_{H}$$

Forward propagation from the hidden layer to the FC layer in the generator:

(7) $$X_{k}^{FC,G}=\sum_{j=1}^{MN_{h}}W_{jk}^{\left(2\right),G}{X_{j}^{Hidden,G}}^{\prime }+b_{k}^{\left(2\right),G}$$

where $N_{H}$ denotes the length of each channel in the hidden layer.

To reduce the number of parameters and make training with small samples more stable, the FC layer nodes are grouped. Forward propagation from the FC layer to the output layer in the generator is as follows:

(8) $$X_{im}^{FC,G}\to X_{j}^{FC,G},\;j=i+(m-1)N_{FC}$$

(9) $$Y_{im}^{G}=\sum_{j=1}^{N_{FC}}W_{jim}^{(3),G}X_{im}^{FC,G}+b_{im}^{(3),G}$$

where $N_{FC}$ is the length of the FC layer. Group $Y_{j}^{G}$ into multiple channels as $Y_{im}^{G}$.

For the discriminator, the forward propagation from the input layer to the hidden layer is the same as that of the generator. The conditional value is fused with the hidden layer output before it is passed to the FC layer. Forward propagation from the hidden layer to the FC layer is as follows:

(10) $$X_{k}^{FC,D}=\sum_{j=1}^{MN_{h}+1}W_{jk}^{\left(2\right),D}{X_{j}}^{Hidden,D}+b_{k}^{\left(2\right),D}$$

Forward propagation from the FC layer to the output layer in the discriminator:

(11) $$Y^{D}=\sum_{k=1}^{N_{FC}}W_{k}^{(3),D}X_{k}^{FC,D}+b_{k}^{(3),D}$$

(12) $$P=Sigmoid(Y^{D})$$

The losses of the discriminator and generator are commonly expressed by the following equations:

(13) $$L_{D}=-\log P_{real}-\log(1-P_{fake})$$

(14) $$L_{G}=-\log P_{fake}$$

where $P_{real}$ and $P_{fake}$ is the probability of judging whether the real and synthesized data is true or false.

The loss values of multiple samples of batch n are averaged to obtain ${\bar{L}}_{D}$ and ${\overset{̿}{L}}_{G}$. Backpropagation denotes the process of updating the network parameters in accordance with the computed averaged loss [27].

Update the parameters by subtracting the gradients corresponding to the generator or the discriminator:

(15) $$W^{*}(n+1)=W^{*}(n)-\eta \frac{\partial \bar{L_{*}}}{\partial W^{*}}$$

(16) $$b^{*}(n+1)=b^{*}(n)-\eta \frac{\partial \bar{L_{*}}}{\partial b^{*}}$$

where η is the learning rate (η=0.001). 

The above steps are repeated until ${\bar{L}}_{D}$ and ${\bar{L}}_{G}$ converge and attain their minimum values.

#### 2.2.2. Data Perturbation

Only a very limited number of samples are available, which makes the model prone to overfitting and may limit its generalization ability. To enhance sample diversity, the output layer of the generator is perturbed in real time during the training process, leveraging the wavelet characteristics. Perturbing the data using the CDF (Cumulative Distribution Function) enhances data diversity while preserving the original statistical characteristics [28].

First, the probability distribution within the wavelet domain is obtained:

(17) $$f(j,i)=\frac{{\left|\varphi_{j,i}\right|}^{2}}{\sum_{i}\sum_{j}{\left|\varphi_{j,i}\right|}^{2}}$$

The cumulative probability distribution function is defined as:

(18) $$F(j,i)=\sum_{i}\sum_{j}f(j,i)$$

For each sampling, a random number u~U(0,1) is generated, and the corresponding index is found via:

(19) $$\left(j^{*},i^{*})=\arg\underset{a^{*},b^{*}}{min}\{F(j^{*},i^{*})\geq u\right\}$$

We add Gaussian noise to the wavelet coefficients in $\left(j^{*},i^{*}\right)$.

(20) $$\varphi_{j^{*},i^{*}}=\alpha \varphi_{j^{*},i^{*}}+\varepsilon ,\;\;\varepsilon \sim N(0,\sigma^{2})$$

where α>0 is an amplitude scaling factor used to control the perturbation strength of the selected coefficient. In this study, α was set close to unity (α∈[0.95,1.05]) to introduce mild amplitude perturbations.

And σ is the standard deviation of the Gaussian noise, which is obtained by the following formula:

(21) $$\sigma =\beta \sqrt{\frac{1}{J\times \sum_{j}^{J}N_{j}}\sum_{i}\sum_{j}{\left|\varphi_{j,i}\right|}^{2}}$$

where β represents the ratio of noise intensity to signal energy. Based on preliminary testing and common practice in signal perturbation, β was selected in the range of 0.01–0.1 to introduce sufficient variability.

The parameters α and β were selected empirically based on preliminary testing and prior experience with wavelet-based signal perturbation. Their values were chosen to introduce sufficient variability while preserving the overall amplitude, energy distribution, and dominant temporal characteristics of the original impact signals. Within the selected ranges, no noticeable degradation in signal quality or model performance was observed.

By perturbing high-energy wavelet coefficients with higher probability, the main signal features are effectively diversified, while low-energy coefficients remain unchanged, thereby preventing the introduction of physically meaningless noise.

### 2.3. Prediction Model

Gaussian Process Regression (GPR) is employed to fuse both test and simulation data, enabling prediction on new inputs. GPR performs well on small- to medium-sized datasets and is capable of capturing the nonlinear influence of input features on the outputs [29,30]. When the input dimensionality is too high, the computation and inversion of the kernel matrix can become extremely costly, making it difficult for the model to effectively fit data. Therefore, for high-dimensional signals, it is usually necessary to first perform dimensionality reduction before applying GPR.

The training set input consists of a combination of the test input load characteristic and the augmentation load signal characteristic. The training set output corresponds to the injury indices evaluated by the Hybrid III 50th ATD physical tests and its numerical simulations. It is expressed as:

(22) $$X=[X^{TST,1},\ldots ,X^{TST,n_{1}},X^{SIM,1},\ldots ,X^{SIM,n_{2}}]$$

(23) $$Y=[index^{TST,1},\ldots ,index^{TST,n_{1}},index^{SIM,1},\ldots ,index^{SIM,n_{2}}]$$

$X^{TST}$ is the test input dataset, $X^{SIM,1}$ is the simulation input dataset, $n_{1}$ is the number of test dataset, and $n_{2}$ is the number of simulation dataset.

For the wavelet coefficients at the j-th scale, the energy is defined as the sum of squares:

(24) $$E_{t}^{j}=\sum_{n=1}^{N_{j}}\varphi_{j,t}^{2}(n)$$

Compute the energy of the wavelet coefficients at each scale to obtain a new curve and we refer to this as the wavelet scale energy curve $\varepsilon_{t}$:

(25) $$\varepsilon_{t}={\left\{E_{t}^{j}\right\}}_{j=1}^{N_{j}}$$

where *t* denotes the index of the *t*-th sample.

However, the dimensionality of the energy distribution is still relatively high for GPR. Principal component analysis (PCA) is applied to the energy distribution curve to reduce dimensionality and extract the dominant components of the data [31]. The advantage of PCA is that the first few principal components usually explain most of the data variance, allowing the original data to be approximately reconstructed with only a small number of components.

The implementation process of PCA is as follows:

To reduce the magnitude differences in the data, $E^{t}$ was log-transformed.

(26) $$E_{log}^{t}=ln(1+E^{t})$$

Mean-centering is performed for each dimension:

(27) $${\bar{E}}_{j}=\frac{1}{n}\sum_{t=1}^{n}E_{j,log}^{t}$$

And subsequently obtain the centered matrix:

(28) $${\tilde{E}}_{tj}=E_{j,log}^{{\bar{t}}_{j}}-{\bar{E}}_{j}$$

The covariance matrix C is of size M×M.

(29) $$C=\frac{1}{M-1}{\tilde{E}}^{T}\tilde{E}$$

Compute the eigenvalues and eigenvectors of the covariance matrix C:

(30) $$Cv_{l}=\lambda_{l}v_{l}$$

(31) $$\lambda_{1}\geq \lambda_{1}\geq \lambda_{3}\geq 0$$

where $\lambda_{l}$ and $v_{l}$ denote the eigenvalue and eigenvector of the covariance matrix C, respectively.

Each curve is projected onto the first three principal components:

(32) $$X_{l}^{t}=(E_{log}^{t}-\bar{E}{)}^{T}v_{l},\;\;\;l=1,2,3$$

The projections of n samples are then assembled to form the input set.

(33) $$X=[X^{1},X^{2},\ldots ,X^{n}]$$

The dimensionality of each data sample is reduced to 4.

Assume that the output follows a Gaussian distribution:

(34) $$Y=f(X)\sim N(0,K(X,X^{T})+\sigma_{n}^{2}I)$$

(35) $$K(X,X^{T}{)}_{ij}=k(X_{i},X_{j})$$

where $\sigma_{n}^{2}I$ denotes the observation noise, ensuring numerical stability of the covariance matrix. $K(X,X^{T})$ is the covariance matrix. The kernel function $k(X_{i},X_{j})$ quantifies the similarity between two vectors. The commonly used formula for the multi-dimensional RBF kernel:

(36) $$\left\{\begin{array}{c} k(X_{i},X_{j})=\sigma_{f}^{2}\exp(-\frac{1}{2}(X_{i}-X_{j}{)}^{T}\Lambda^{-1}(X_{i}-X_{j}))\\ \Lambda =diag(l_{1}^{2},l_{2}^{2},\ldots ,l_{M}^{2}) \end{array}\right.$$

where $\sigma_{f}^{2}$ represents the signal variance, $l_{i}$ denotes the length scale for each input dimension; $\sigma_{f}^{2}$, $\sigma_{n}^{2}$ and $l_{i}$ are hyperparameters.

Marginal likelihood is expressed as:

(37) $$p(Y|X,\theta )=\frac{1}{(2\pi {)}^{n/2}{\left|K^{\prime }\right|}^{1/2}}\exp(-\frac{1}{2}Y^{T}K^{\prime -1}Y)$$

(38) $$K^{\prime }=K+\sigma_{n}^{2}I$$

where $\theta =\{\sigma_{f},\sigma_{n},l\}$ is the set of hyperparameters.

The set of hyperparameters that maximizes the log marginal likelihood is determined via gradient descent.

(39) $$\theta^{*}=\arg\underset{\theta }{max}\log p(Y|X,\theta )$$

The resulting model combines the test signals and the simulated signals, establishing the mapping relationship between the load signals and the injury indices.

The GPR model was implemented using MATLAB (R2024a, MathWorks, Natick, MA, USA) fitrgp function with RBF kernel. The hyperparameters, including length scale, signal variance, and noise variance, were automatically optimized during training.

### 2.4. Injury Indices

Lower limb and spinal compressive injuries are among the most common occupant injuries caused by landmine blasts. The following introduces the injury indices used to assess injury at these two body regions. The aim of our study is to reduce the injury indices as much as possible through optimization.

#### 2.4.1. Lower Limb Injuries

The tibial force should be filtered according to SAE J211-1 [32] using a Channel Frequency Class (CFC) of 600. In lower limb injury evaluation protocols, tibial axial force (Fz) serves as the critical measurement parameter, with the peak compressive value being the key determinant [33].

(40) $$Pass:\left|Fz_{p}\right|<Fz_{c}$$

(41) $$Fail:\;\left|Fz_{p}\right|\geq Fz_{c}$$

$Fz_{p}$ is the peak value (maximum amplitude) in the compression part of the axial force signal in the lower tibia and where $Fz_{c}$ is the critical limit value of the tibial compression force.

The AEP-55 specifies a lower-limb tolerance value of $Fz_{c}=5.4\;kN$, corresponding to a 10% risk of AIS 2+ injury [34].

#### 2.4.2. Spine Injuries

The pelvic acceleration was filtered according to SAE J211-1 using CFC 1000. Payne and Stech introduced the Dynamic Response Index (${DRI}_{z}$) as a simplified biomechanical model to represent human body dynamics, employing a single mass-spring-damper system [35]. The ${DRI}_{z}$ serves as a measure for assessing injuries caused by axial compression in the thoracolumbar spine. It is a dimensionless parameter that correlates with spinal deflection, which represents the degree of compression. This deflection is determined by the response of a second-order system, where the input is the vertical acceleration of the pelvis.

The equation of motion for this model is as follows:

(42) $$\ddot{z}\left(t\right)=\ddot{\delta }+2\zeta \omega_{n}\dot{\delta }+\omega_{n}^{2}\delta$$

where $\ddot{z}\left(t\right)$ is the acceleration in the vertical direction [$m/s^{2}$]; $\delta =\zeta_{1}-\zeta_{2}$ (when δ>0) is the deflection (compression) of the system; $\zeta =\frac{c}{2m\omega_{n}}$ is the damping coefficient (0.224), and $\omega_{n}=\sqrt{\frac{k}{m}}$ is the circular frequency (52.9 rad/s).

The DRIz is calculated from the maximum compression $\delta_{max}$ and the gravity acceleration g (9.81 $m/s^{2}$):

(43) $$DRIz=\frac{\omega_{n}^{2}\delta_{max}}{g}$$

The AEP-55 specifies a spinal injury tolerance value of DRIz=17.7.

## 3. Data Acquisition

### 3.1. Experiments and Simulations

**Figure 3:**
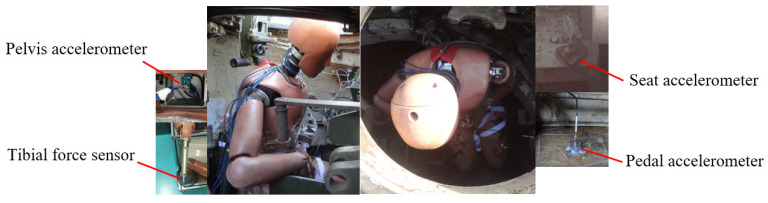
Military vehicle underbelly blast test setup.

As shown in Figure 3, the Hybrid III 50th ATD physical model was positioned inside a computational model of a vehicle, and an explosive was located directly beneath the vehicle’s body on the concrete ground. The exact explosive equivalent is not publicly disclosed for security reasons, but the detonation scenario was designed to represent a typical underbody blast threat. The seat was rigidly attached to the vehicle sidewall without any vibration isolation devices or occupant restraint systems, thereby representing a worst-case loading condition. Acceleration sensors were installed at the pedal and seat locations, while a pelvis accelerometer and a tibial force sensor were embedded in the ATD to record the dynamic responses.

**Figure 4:**
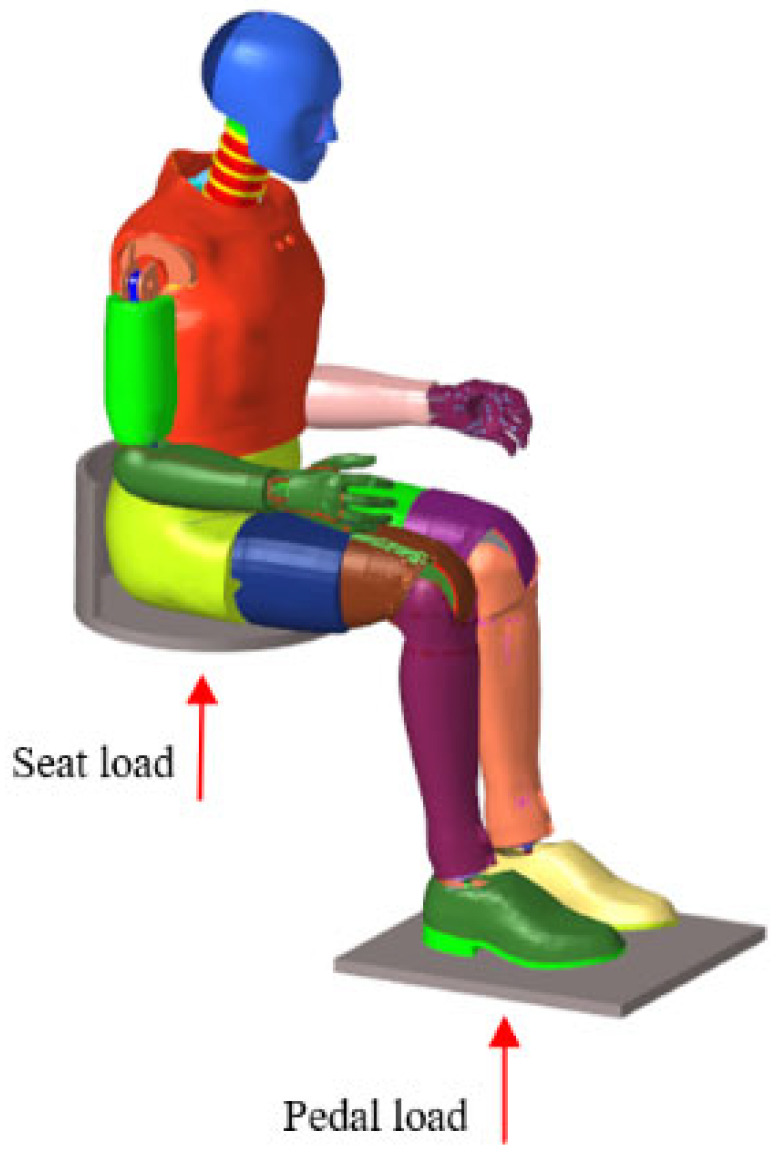
Finite element model set up.

The sensor-measured signals were imposed on the FE model at the respective sensor locations, ensuring that the input loading replicates the measured conditions, as illustrated in Figure 4. Experimentally measured accelerations at the pedal and seat were imposed on the corresponding rigid bodies within the FE model to reproduce the blast loading environment. The contact between the pedal/seat and the dummy was defined using the keyword CONTACT_AUTOMATIC_NODES_TO_SURFACE. The Hybrid III 50th FE dummy was optimized following the approach described by Suhaimi [6]. Section 3.3 compares the dynamic response curves obtained from the FE dummy with those from the physical Hybrid III dummy.

To ensure the reproducibility of the proposed method, the typical hyperparameter settings used in training the GAN model are summarized in Table 1. These settings were applied to all experiments unless otherwise specified.

### 3.2. Blast Impact Signal Analysis

The effects of landmines on armored vehicles can be broadly categorized into local effects and global effects. Local effects refer to local structures and are often characterized by high-frequency vibrations. Global effects refer to the overall response of the entire vehicle or its large-scale structures’ deformation to an explosion, characterized by low-frequency vibrations.

**Figure 5:**
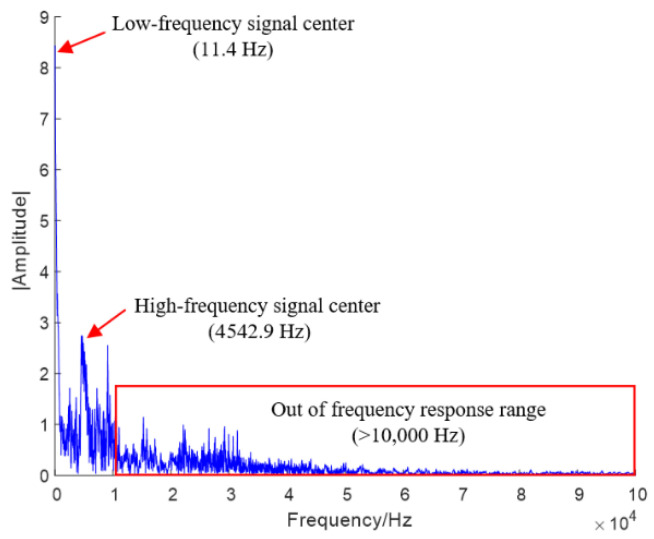
Frequency spectrum of Test 1 pedal impact signals.

The sensor employed for the test load signal is the Kistler 8743 accelerometer (Kistler Group, Winterthur, Switzerland), with a measurement range of ±100,000 g. According to the manual, the frequency response range of the sensor is 1–10,000 Hz. Therefore, frequency components above $10^{4}$ Hz are not considered in this study. The test load signals were acquired at a sampling frequency of $10^{5}$ to prevent aliasing and subsequently downsampled to $10^{4}$ during signal processing. Figure 5 shows the frequency spectrum of an underbody blast impact signal. Analysis of the frequency spectrum reveals two peaks within the sensor’s frequency response range: one at 11.4 Hz, identified as the center of the low-frequency component, and another at 4542.9 Hz, identified as the center of the high-frequency component. This imposes demands on methods capable of distinguishing different frequency signal components. The complexity of the signals increases processing difficulty.

### 3.3. Model Performance Evaluation

The degree of agreement between the actual and simulated signals is assessed using ISO 18571. According to ISO 18571 [36], the evaluation is performed using a weighted combination of the corridor score, phase score, magnitude score, and slope score. Assume the test data are represented by x(t) (reference curve), while the simulation data are represented by y(t) (comparison curve).

Two corridor areas are defined around the reference curve, with the simplest constant-width definition shown below.

Inner corridor:

(44) $$\delta_{i}(t)=a_{0}Y_{norm}$$

Outer corridor:

(45) $$\delta_{o}(t)=b_{0}Y_{norm}$$

The standard specifies $a_{0}=0.05$, $b_{0}=0.5$. $Y_{norm}$ is defined as the reference amplitude employed in the calculation of corridors:

(46) $$Y_{norm}=\max(\min(x(t)),\max(x(t)))$$

The corridor rating is first computed at each time step $t_{i}$, followed by averaging to obtain the final score.

(47) $$E_{ci}=\left\{\begin{array}{cc} 1 & if\;\left|y(t_{i})-x(t_{i})\right|<\delta_{i}(t)\\ {\left(\frac{\delta_{o}(t)-\left|y(t_{i})-x(t_{i})\right|}{\delta_{o}(t)-\delta_{i}(t)}\right)}^{k_{c}} & k_{c}=2\\ 0 & if\;\left|y(t_{i})-x(t_{i})\right|>\delta_{o}(t) \end{array}\right.$$

(48) $$E_{c}=\frac{\sum_{i=1}^{N}E_{ci}}{N}(0\leq E_{ci}\leq 1)$$

N is the number of sampling points, $E_{ci}=0$ indicates the correlation is poor and $E_{ci}=1$ indicates the correlation is good.

The phase shift score $E_{p}$ is then calculated for each time shift using the following formula:

(49) $$l=\varepsilon_{pmax}N$$

where $\varepsilon_{pmax}$ is the maximum number of time-shifts.

Shift the simulation signal to the right by m samples and compare it with the test signal.

(50) $$P_{L}\left(m\right)=\frac{\sum_{i=0}^{l-1}\left[(y\left(t_{start}+\left(m+i\right)\Delta t\right)-\bar{y}(t))(x\left(t_{start}+i\Delta t\right)-x(t))\right]}{\sum_{i=1}^{l-1}[y\left(t_{start}+\left(m+i\right)\Delta t\right)-\bar{y}\left(t\right){]}^{2}\sum_{i=0}^{l-1}[x\left(t_{start}+i\Delta t\right)-\bar{x}(t){]}^{2}}$$

where $t_{start}$ is the start time for the signal comparison.

Shift the test signal to the right by m samples and compare it with the simulation signal.

(51) $$P_{R}\left(m\right)=\frac{\sum_{i=0}^{l-1}\left[(y\left(t_{start}+i\Delta t\right)-\bar{y}(t))(x\left(t_{start}+(m+i)\Delta t\right)-x(t))\right]}{\sum_{i=1}^{l-1}[y\left(t_{start}+i\Delta t\right)-\bar{y}\left(t\right){]}^{2}\sum_{i=0}^{l-1}[x\left(t_{start}+(m+i)\Delta t\right)-\bar{x}(t){]}^{2}}$$

(52) $$l_{\varepsilon }=\arg\underset{m}{max}(\max(P_{L}(m),P_{R}(m)))$$

$l_{\varepsilon }$ is the value of m that gives the best alignment.

The phase shift score is then calculated according to the following equation:

(53) $$E_{p}=\left\{\begin{array}{cc} 1 & if\;l_{\varepsilon }=0\\ {\left(\frac{\varepsilon_{pmax}N-l_{\varepsilon }}{\varepsilon_{pmax}N}\right)}^{k_{p}} & k_{p}=1\\ 0 & if\;l_{\varepsilon }>\varepsilon_{pmax}N \end{array}\right.$$

$E_{m}$ is computed from the residual overlap of the curves after the time shift determined by $l_{\varepsilon }$. $y^{ts}(t)$ is the truncated and shifted part of the simulation curve, while $x^{t}(t)$ is the remaining truncated part of the test curve. For $x^{t}(t)$ and $y^{ts}(t)$, DTW is implemented. The distance between each point of the two sequences is calculated to form a cost cumulative matrix.

(54) $$d(i,j)=(x(i)-y(j){)}^{2}$$

The DTW-aligned signal is longer ($n\leq n_{new}\leq 2n-1$) and most points follow the optimal warping path. The amplitude error is computed after alignment along the optimal warping path.

(55) $$\varepsilon_{mag}=\frac{{\left\|y^{ts+d}(t)-x^{t+d}(t)\right\|}_{1}}{{\left\|x^{t+d}(t)\right\|}_{1}}$$

The magnitude score is defined as:

(56) $$E_{m}=\left\{\begin{array}{cc} 1 & if\;\varepsilon_{mag}=0\\ {\left(\frac{\varepsilon_{m}-\varepsilon_{mag}}{\varepsilon_{m}}\right)}^{k_{m}} & {k_{m}=1,\varepsilon }_{m}=0.5\\ 0 & if\;\varepsilon_{mag}>\varepsilon_{m} \end{array}\right.$$

Compute the derivatives of the two curves using the central difference method. Before calculating $E_{s}$, the curve was smoothed using a 9-point moving average. At the endpoints, a reduced window of 1 to 5 points was used. After smoothing, the curves $x^{t+d}$ and $y^{ts+d}$ were derived. The slop error is computed:

(57) $$\varepsilon_{slope}=\frac{{\left\|y^{ts+d}(t)-x^{t+d}(t)\right\|}_{1}}{{\left\|x^{t+d}(t)\right\|}_{1}}$$

The slope score is defined as:

(58) $$E_{s}=\left\{\begin{array}{cc} 1 & if\;\varepsilon_{slope}=0\\ {\left(\frac{\varepsilon_{s}-\varepsilon_{slope}}{\varepsilon_{s}}\right)}^{k_{s}} & {k_{s}=1,\varepsilon }_{s}=2.0\\ 0 & if\;\varepsilon_{slope}>\varepsilon_{s} \end{array}\right.$$

The total ISO score is calculated as a weighted sum of the four component ratings:

(59) $$R=W_{c}E_{c}+W_{p}E_{p}+W_{m}E_{m}+W_{s}E_{s}$$

The ISO 18571 component weights were selected with specific consideration of finite element model (FE model) validation. In FE model-based injury prediction, preserving the overall temporal correlation between loading signals is critical, as it directly influences the global dynamic response of the model. Therefore, a higher weight was assigned to the correlation component ($W_{c}=0.4$) to prioritize consistency in overall response trends between FE model simulations driven by original and synthesized signals. The phase, magnitude, and shape components were assigned equal but lower weights ($W_{p}=0.2$, $W_{m}=0.2$, $W_{s}=0.2$) to constrain localized discrepancies while preventing any single feature from disproportionately affecting the assessment.

### 3.4. Results and Discussion

**Figure 6:**
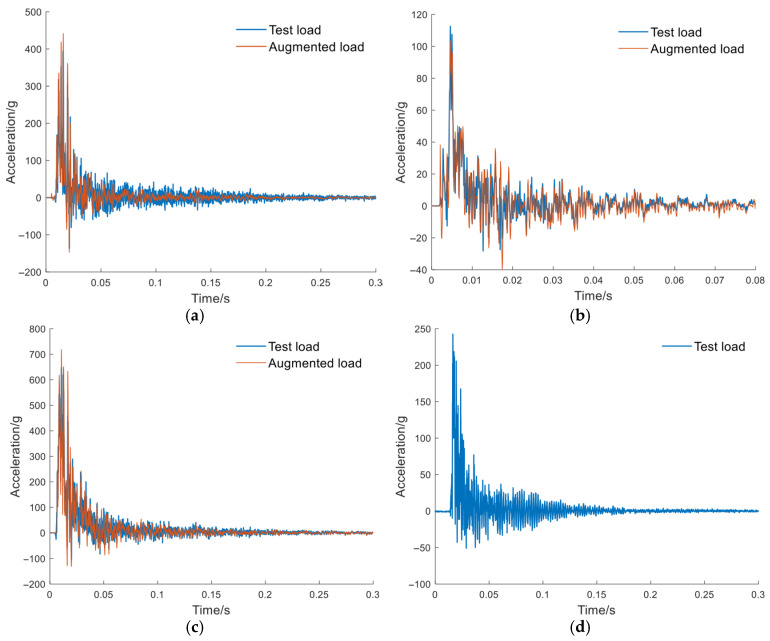
Pedal load signals: measured signals and augmented signals, with the augmented signals matching the amplitude of the measured signals. (**a**) Test #1; (**b**) Test #2; (**c**) Test #3; (**d**) Test #4.

**Figure 7:**
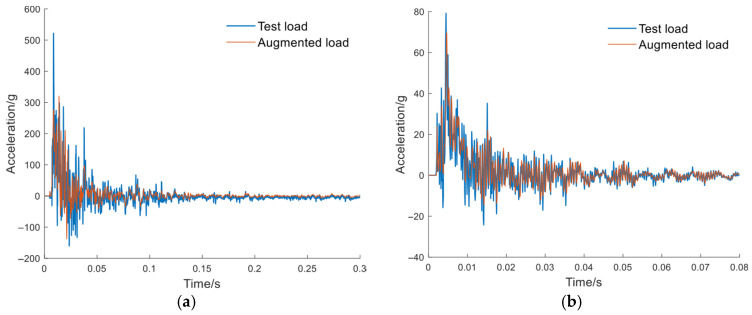

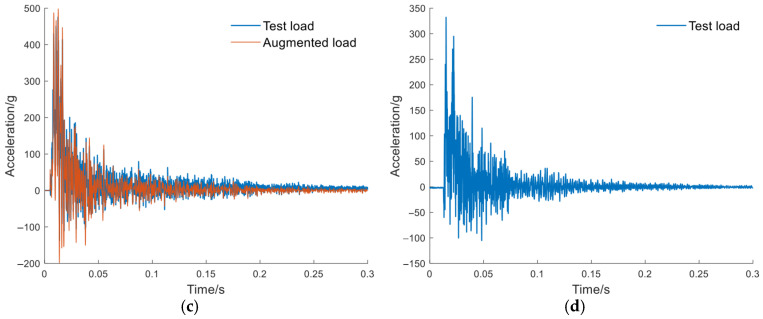
Seat load signals: measured signals and augmented signals, with the augmented signals matching the amplitude of the measured signals. (**a**) Test #1; (**b**) Test #2; (**c**) Test #3; (**d**) Test #4.

**Figure 8:**
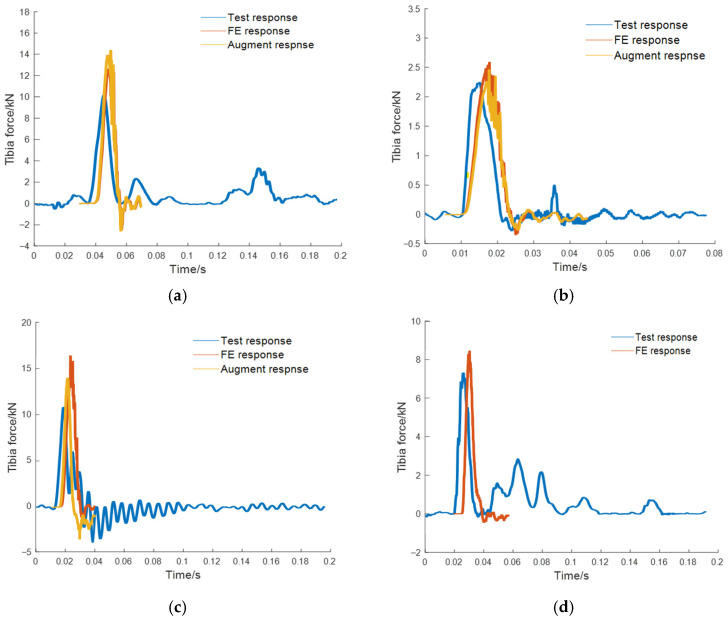
Comparison of the tibial force from the test, the FE tibial force under the original test pedal load, and the FE tibial force under the amplitude-matched augmented pedal load. (**a**) Test #1; (**b**) Test #2; (**c**) Test #3; (**d**) Test #4.

**Figure 9:**
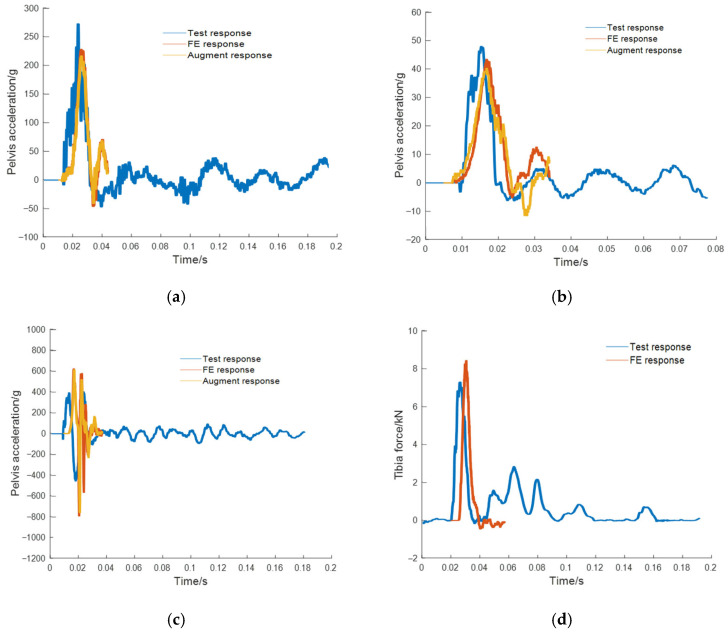
Comparison of the pelvis acceleration from the test, the FE pelvis acceleration under the original test seat load, and the FE pelvis acceleration under the amplitude-matched augmented seat load. (**a**) Test #1; (**b**) Test #2; (**c**) Test #3; (**d**) Test #4.

The load signals from experiments were recorded using onboard accelerometers. A total of eight datasets were employed for training the model, among which four-fold cross-validation was performed. Figure 6 and Figure 7 show the acceleration at the pedal and seat monitoring locations. To demonstrate the effectiveness of the proposed CGAN-based load signal augmentation, a subset of representative signals was selected for visualization. The synthesized accelerations for the pedal and the seat under the same amplitude were compared. The synthesized data were employed as loading inputs to the FE model to evaluate occupant injury, with the quality of the synthesized data assessed based on the accuracy of FE-predicted biomechanical responses. To facilitate a direct comparison, the synthesized signal’s amplitude was normalized to match that of the original signal. Using the measured load signals as inputs, the FE model simulated the response curves, which were then compared with the experimental results. The tibial axial force is presented in Figure 8, while the pelvic acceleration is shown in Figure 9. Table 2 presents the ISO scores for the simulated and experimental responses. The simulated response for Test 2 showed poor agreement, with ISO scores below 0.5, likely due to the load impact exceeding the range for which the FE model was optimized. Consequently, simulated data involving particularly high loads were excluded. In addition, Table 1 shows that the ISO scores of the FE responses driven by the synthesized signals closely match those driven by the original test load signal, demonstrating the reliability and effectiveness of the generated data for the intended application.

**Figure 10:**
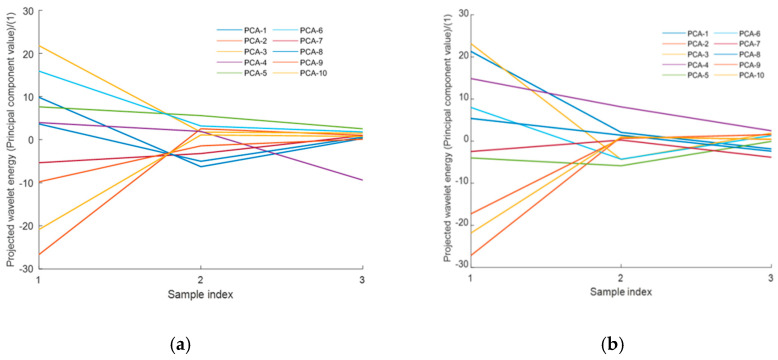
First three principal components extracted from the wavelet scale energy curves by PCA: (**a**) Pedal load signals and (**b**) seat load signals. (The first three principal components account for no more than 76.2% of the variance for pedal and no more than 72.8% for seat).

Additional sets of augmented load signals with varying amplitudes were generated by CGAN by adjusting c in data-augmentation model. PCA was performed on the combined data, and the eigenvalues of the first three components were used as inputs to the GPR model, with the corresponding injury indices as outputs (Table 3, Figure 10).

The predictive performance of the GPR model was evaluated using Leave-One-Out Cross-Validation (LOOCV). In each iteration, one sample was held out as the test set while the remaining samples were used for training. The maximum prediction error of the training set across all iterations was used to assess the model’s fitting performance, while the test set error was computed to evaluate predictive performance. To further evaluate generalization, data from Test 4 were excluded from serving as real data in the augmentation model. The relative errors (RE) are summarized in Equation (60). RE is expressed as:

(60) $$RE=\frac{\left|{Index}_{pred}-{Index}_{test}\right|}{\left|{Index}_{test}\right|}\times 100\%$$

${Index}_{pred}$ is the predicted injury index and ${Index}_{test}$ is the test injury index.

The prediction errors of the four datasets as training and testing sets are shown in Table 4. For the training set, the maximum error among all cycles was reported below 10%, demonstrating strong model fit. Compared with FE model predictions, the GPR prediction errors on the testing set were lower, indicating a clear improvement in predictive capability.

## 4. Case Study: Armored Vehicles Underbody Structure Optimization

In this study, FEA was used to preliminarily assess the feasibility of the proposed method. However, due to the simplified assumptions in the vehicle FE model and the potential influence of numerical errors, the accuracy of the method cannot be fully guaranteed. Therefore, future research should focus on validating its performance under practical conditions.

### 4.1. Effect of Structure Geometry

The V-hull structure is a passive protection technology specially designed to resist bottom explosion threats such as landmines and improvised explosive devices (IEDs), and is widely used in military armored vehicles. The bottom adopts an inclined V-hull structure configuration, guiding the explosion shock wave to both sides to reduce the direct impact on the vehicle body. This section focuses on quantifying the reduction in occupant lower-limb injuries achieved by the V-hull structure compared to the flat-hull structure.

Trajkovski et al. demonstrated that the V-hull significantly reduces blast-induced human injury compared to a flat-hull, based on comparative analyses of peak floor acceleration and kinetic energies [37]. While blast-induced human injury depends not only on the magnitude of the impact but also on its duration of action, such an analysis is insufficient.

**Figure 11:**
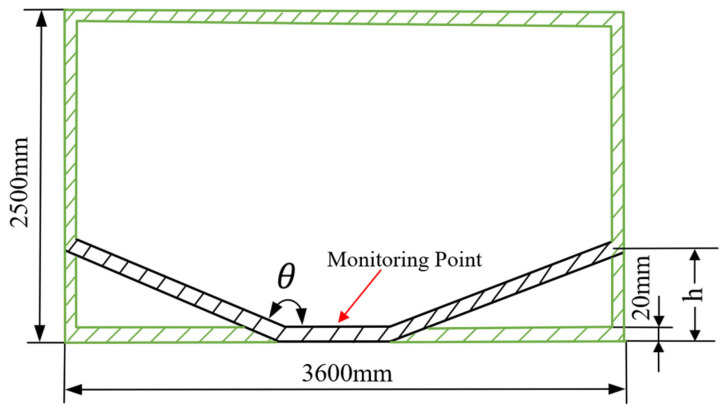
Dimensions of the flat-hull and V-hull configurations of the heavy armored vehicle body model θ.

Vehicle body models were established in the paper: a flat-hull structure and a V-hull structure. The model utilized Hardox 400 steel with identical masses of 14,000 kg after counterweight adjustment. The thickness of the chassis is 20 mm. Charges with TNT equivalents of 6 kg, 8 kg, and 10 kg were detonated at a standoff distance of 500 mm from the bottom plate. Other dimensions of the structure are shown in Figure 11. The FE model parameters were configured with reference to the work of Glavšić and Elek [38]. The V-hull structure has θ (hull angle) of 150° and *h* (hull depth) of 800 mm. The monitoring point was positioned on the upper surface of the armored vehicle chassis, directly above the detonation point.

**Figure 12:**
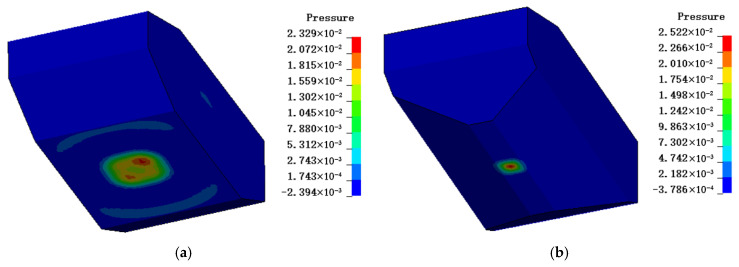
Pressure contour plot of heavy armored vehicle body under explosion: (**a**) Flat-hull and (**b**) V-hull.

Figure 12 presents the pressure contour plots from finite element analysis, comparing flat-hull and V-hull structures under blast loading. It can be seen that under the same maximum pressure, the pressure concentration area of flat-hull is larger than that of V-hull. Nevertheless, the contour plot cannot fully reflect the injury status of occupants. The following is an analysis based on the predictive model.

**Figure 13:**
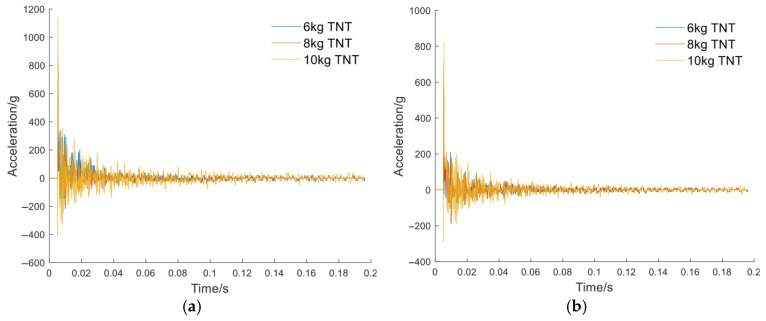
Monitoring point accelerations for different TNT equivalent charges: (**a**) Flat-hull and (**b**) V-hull.

**Figure 14:**
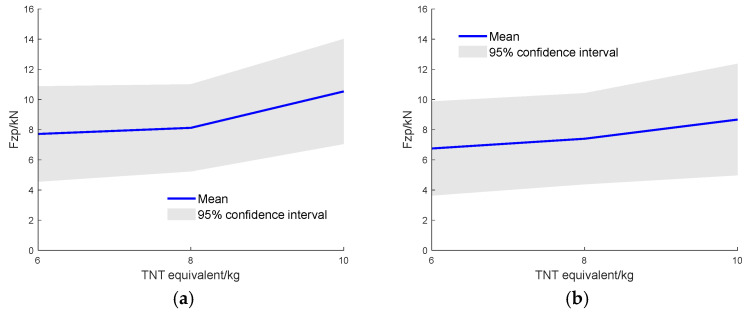
GPR-predicted lower limb injury indices for different TNT equivalent charges: (**a**) Flat-hull and (**b**) V-hull.

Figure 13 represents the accelerations at the monitoring points of the FE model for the flat-hull and V-hull structures under three equivalent TNT charges. After extracting features from the scaled energy curves using PCA, they were input into the pre-trained GPR model. Then, the curves of the predicted mean axial tibial compression force versus TNT equivalent charge for the two types of structural geometry were obtained by the model, along with the 95% confidence intervals, as shown in Figure 14.

According to the predicted mean values, at TNT equivalent charges of 6 kg, 8 kg, and 10 kg, the V-hull structure reduced the lower-limb injury index by 11.7%, 12.4%, and 17.4%, respectively, compared with the flat-hull structure. The influence of structural geometry becomes more pronounced as the TNT equivalent charge increases. Compared with the lower-limb injury threshold of 5.4 kN, the predicted peak Fz values (7.9–10.1 kN for the flat-bottom design and 6.9–8.8 kN for the V-shaped design) suggest that both configurations could cause injury under high loading conditions. Nevertheless, the V-hull structure exhibits noticeably lower lower-limb forces, implying improved energy absorption and enhanced safety performance. Furthermore, the relatively narrow confidence intervals indicate that the training data adequately cover the input space. In contrast, when the confidence interval becomes wider, additional experimental data or data augmentation will be required in future work to improve model reliability.

### 4.2. Effect of Structure Parameters

**Figure 15:**
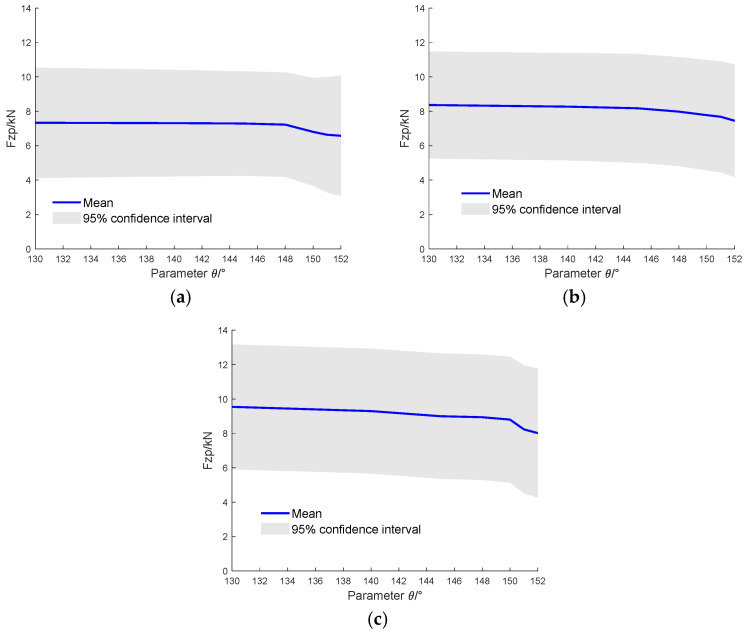
GPR-predicted lower limb injury indices with fixed h and varying θ: (**a**) 6 kg TNT equivalent charge (**b**) 8 kg TNT equivalent charge and (**c**) 10 kg TNT equivalent charge (The solid line represents the mean prediction, and the shaded area indicates the 95% confidence interval).

The purpose of this section is to identify the optimal structural parameters. The vehicle model parameters were varied, and the accelerations at the monitoring points were employed as inputs to the pre-trained GPR. The selected parameter range is just enough to maintain the V-hull geometry. Figure 15 illustrates the case with h fixed at 800 mm while varying the range of θ. The curve indicates that as θ decreases, the tibial maximum force is reduced. The reductions were 10.3% for 6 kg, 10.9% for 8 kg, and 15.9% for 10 kg TNT equivalent charges. The curve exhibits a noticeably faster decline beyond 148°.

**Figure 16:**
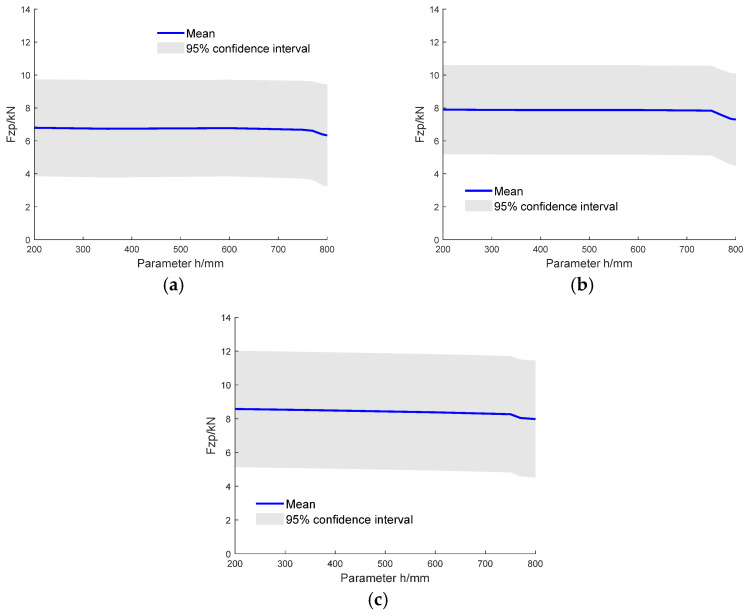
GPR-predicted lower limb injury indices with fixed θ and varying h: (**a**) 6 kg TNT equivalent charge (**b**) 8 kg TNT equivalent charge and (**c**) 10 kg TNT equivalent charge (The solid line represents the mean prediction, and the shaded area indicates the 95% confidence interval).

Figure 16 illustrates the case with θ fixed at 150° while varying the range of h. The curve indicates that as the h increases, the tibial maximum force reductions were 7.9% for 6 kg, 7.6% for 8 kg, and 6.9% for 10 kg TNT equivalent charges. The curve exhibits a noticeably faster decline beyond 150 mm. When the other parameter is held constant, increasing θ or h both lead to a reduction in the lower-limb injury index. Adjusting θ has a more significant effect than adjusting h, suggesting that angle adjustment should take priority, with hull depth adjustment serving as a supplement.

## 5. Discussion

The current framework directly concatenates experimental and simulated data into a single training set. While this approach allows efficient use of limited data, it neglects the source-dependent uncertainties and potential discrepancies between simulation and experiment. As a result, the model may inherit systematic biases from either data source, and its generalization performance could be affected when the source distributions differ. Saadallah et al. introduced three data fusion strategies: data-level fusion, model-level fusion, and level choice decision that selects the fusion level according to the bias-variance trade-off [39]. Xiong proposed a statistical data fusion framework integrating observations and model hindcasts to estimate environmental hazard footprints, introducing a structured Gaussian process discrepancy and validating the method on windstorm Imogen [40]. In this study, experimental and simulation data differ in quality, bias, and uncertainty. A fixed fusion strategy would ignore these differences, causing the model to be affected by the lower-quality data. Fei proposed an adaptive multi-source fusion framework based on Dempster-Shafer theory and validated it using a disaster case, demonstrating dynamic adjustment of factor weights and fusion levels in emergencies [41]. Yin developed a global Kalman–LSTM-based adaptive fusion method for UAV target tracking, validated in both simulation and real flight experiments [42]. Yue introduced a dual-attention and deep reinforcement learning-based adaptive fusion method for cross-modal retrieval, surpassing traditional neural models in performance [43].

Building on these ideas, future work in this study will focus on an adaptive Bayesian fusion GPR framework, in which each data source is assigned a weight reflecting its reliability. These weights are adaptively determined based on prediction error, variance for sources with experimental comparisons, while unobserved simulation data and physical measurements are assigned fixed weights, with the physical data given maximum weight. The weights are adaptively updated during the training process. The weighted GPR output can be conceptually represented through Bayesian model averaging as:

$$p\left(Y|X,D\right)=\sum_{i}w_{i}p(Y|X,M_{i})$$

where $M_{i}$ denotes the *i*-th data source and $w_{i}$ is corresponding weight.

This approach enables the fusion of predictive distributions from simulation- and experiment-based models, balancing accuracy and uncertainty across different sources, and effectively leveraging all available information while mitigating the influence of lower-quality data.

In addition, the proposed hybrid modeling approach also shows potential for broader applications. Future studies may extend this methodology to innovative energy-absorbing or damping systems, as well as to various vehicle structures and loading conditions, which would further enhance its generality and engineering relevance.

## 6. Conclusions

To address the trade-off between underbody blast protection optimization experimental costs and data requirements, this study developed a prediction method combining physical testing and digitally driven techniques. A small-scale data augmentation framework based on CGAN was constructed within the wavelet domain for load signal augmentation and an FE model was employed to generate augmented injury indices. During training, real-time perturbations are added to enhance the diversity of the dataset. The primary features of the wavelet scale energy of the load signals were extracted through PCA, and a GPR-based mapping between load signal features and injury indices was established by integrating limited measured data with augmented data. LOOCV demonstrates that the proposed approach achieves better performance than FE simulations. Finally, the trained GPR model was successfully applied to analyze the effects of the armored vehicle underbody structural characteristics on occupant injury in a simulation case.

Below is a summary of final conclusions:

1. According to the ISO score, excessive loads cause significant FE distortion, so data augmentation should exclude extreme impact scenarios. (In the study, the pedal acceleration peak exceeded 600 g, the seat acceleration peak exceeded 400 g, and the tibia force and pelvis acceleration ISO scores were below 0.5). Using LOOCV, the GPR-based prediction model exhibited superior fitting capability, yielding smaller prediction errors for the injury index compared to direct FE application.

2. The trained integrated GPR model was used to optimize the geometry and structural parameters of the vehicle under underbody blast loading. According to the AEP-55 criteria, lower peak tibial axial forces $Fz_{p}$ correspond to a lower probability of lower-limb injury. A set of simulation cases demonstrated that the V-hull reduces the peak tibial axial forces compared with the flat-hull, with the advantage becoming more pronounced as the explosive charge increases. In addition, with the hull depth held constant, increasing the hull angle improves blast resistance, whereas with the hull angle held constant, increasing the hull depth also enhances blast resistance. The effect of varying the hull angle is more pronounced than that of varying the hull depth.

Compared with directly using FE simulations, this method improves prediction accuracy. However, GPR predictions exhibit wide confidence intervals, mainly due to the sparsity of the training data in certain regions of the input space. In this study, only eight physical blast tests were available for model training, which limits the coverage of the design domain. Moreover, although the proposed method demonstrates high performance in the present study, future work should account for occupant restraints, the coupling between the feet and pedals, the interaction between the gluteal region and seat, and validation across different vehicle datasets to ensure a more comprehensive evaluation.

## Figures and Tables

**Table 1:** Network and Training Settings.

Layer	Input Layer	Hidden Layer	FC Layer	Output Layer
Generator neurons	$N_{j}$	$N_{j}/5$	$N_{j}/10$	$N_{j}$
Discriminator neurons	$N_{j}$	$N_{j}/4$	$N_{j}/10$	$N_{j}$
Activation function				Sigmoid
Epochs	1000			
Batch size	4			
Update ratio (Generator: discriminator)	1:2			

Note: $N_{j}$ denotes the input data length at the *j*-th wavelet scale. $N_{1}=3000$ in the paper.

**Table 2:** ISO scores of the FEA response signals.

Test Nos	Tibial Axial Compressive Force (kN)				Pelvis Acceleration (g)			
	FEA with Original Load Signals		FEA with Augmented Load Signals		FEA with Original Load Signals		FEA with Augmented Load Signals	
	Z	($E_{c},E_{p},E_{m},E_{s}$)	Z	($E_{c},E_{p},E_{m},E_{s}$)	Z	($E_{c},E_{p},E_{m},E_{s}$)	Z	($E_{c},E_{p},E_{m},E_{s}$)
1	0.58	(0.49, 0.43, 0.71, 0.79)	0.61	(0.51, 0.50, 0.72, 0.80)	0.61	(0.56, 0.70, 0.59, 0.67)	0.63	(0.55, 0.70, 0.62, 0.72)
2	0.75	(0.73, 0.74, 0.78, 0.80)	0.76	(0.73, 0.71, 0.79, 0.81)	0.69	(0.60, 0.63, 0.87, 0.79)	0.67	(0.62, 0.62, 0.71, 0.76)
3	0.44	(0.41, 0.17, 0.45, 0.78)	0.54	(0.39, 0.53, 0.57, 0.81)	0.42	(0.57, 0.00, 0.37, 0.61)	0.47	(0.55, 0.00, 0.57, 0.66)
4	0.71	(0.70, 0.47, 0.79, 0.87)	-	-	0.54	(0.48, 0.48, 0.56, 0.70)	-	-

**Table 3:** GPR model with PCA-*i* as input and injury indices as output (PCA-*i* represents the principal component derived from the *i*-th dataset. For GPR modeling, the first three principal components were used as input variables).

Injury Indices		Principal Components								
	PCA-1	PCA-2	PCA-3	PCA-4	PCA-5	PCA-6	PCA-7	PCA-8	PCA-9	PCA-10
$Fz_{p}$ (kN)	9.3	2.3	11.1	7.5	7.8	8.2	4.1	7.2	5.2	2.8
DRIz	65.6	6.5	90.6	36.8	12.5	30.1	14.4	20.1	11.8	10.2

**Table 4:** Errors of predicted injury indices.

Test Nos	RE (%)					
	$Fz_{p}$			DRI_z_		
	GPR Model Prediction		FEA	GPR Model Prediction		FEA
	Training (Max)	Testing		Training (Max)	Testing	
1	8.6	12.4	28.1	0.8	14.4	18.5
2	4.3	3.2	13.0	0.4	12.3	17.1
3	8.1	19.3	59.1	5.1	18.2	212.1
4	2.7	4.3	13.3	1.2	13.2	32.5

## Data Availability

The original contributions presented in this study are included in the article. Further inquiries can be directed to the corresponding author.

## Abbreviations

The following abbreviations are used in this manuscript:

CGAN	conditional generative adversarial network
GPR	Gaussian process regression
FE	finite element
CFD	Computational Fluid Dynamics
FEA	finite element analysis
FC	fully connected
DB4	Daubechies 4
PCA	Principal component analysis
CFC	Channel Frequency Class
DRI_z_	Dynamic Response Index
LOOCV	Leave-One-Out Cross-Validation
RE	relative errors
IEDs	improvised explosive devices
